# The role and relationship between programmed death ligand 1 and cytotoxic T lymphocyte-associated antigen-4 immunohistochemical expression in colorectal carcinoma patients: an impact on outcome

**DOI:** 10.3332/ecancer.2021.1323

**Published:** 2021-11-25

**Authors:** Asmaa Shams El Dein Mohameda, Hala Said El-Rebey, Lamia Sabry Abdelsamed AboElnasr, Asmaa Gaber Abdou

**Affiliations:** Department of Pathology, Faculty of Medicine, Menoufia University, Shebein Elkom, 32511, Egypt; a https://orcid.org/0000-0003-0869-6332

**Keywords:** PD-L1, CTLA-4, immune checkpoints, CRC, OS, RFS

## Abstract

**Background:**

Globally, colorectal carcinoma (CRC) is the third most common cancer diagnosed in both men and women. Programmed death ligand 1 (PD-L1) and cytotoxic T lymphocyte-associated antigen-4 (CTLA-4) are immune checkpoints that induce tumour immune escape.

**Aim:**

This study aimed to evaluate the immunohistochemical expression of PD-L1 and CTLA-4 in CRC and their relationship with clinicopathological parameters and survival data.

**Result:**

This study included 103 CRC, 22 adenoma and 21 non-neoplastic specimens. High PD-L1 epithelial expression was in favour of CRC and high-grade dysplastic adenoma compared to normal specimens. High PD-L1 epithelial expression was associated with larger sized tumours, perforation, advanced T stage, infiltrative tumour border configuration (TBC), high tumour budding (TB) score, low tumour-stroma ratio (TSR) and absence of peritumoural lymphocytes. High PD-L1+ tumour infiltrating lymphocytes (TILs) showed an association with absence of perforation, early T stage, pushing TBC, lower TB score, high TSR and presence of peritumoural lymphocytes. High epithelial CTLA-4 expression was in favour of adenocarcinoma, high-grade dysplastic adenoma and low-grade dysplastic adenoma compared to normal specimens. High CTLA-4 epithelial score showed an association with positive lymph nodes (LNs), presence of an infiltrative TBC and absence of peritumoural lymphocytes. Low CTLA-4+ TILs showed a significant association with advanced tumour stage and increased number of positive LNs. Prolonged survival was associated with low epithelial PD-L1 and CTLA-4, high PD-L1+ TILs and high CTLA-4+ TILs. By multivariate Cox regression analysis, PD-L1+ TILs immunoreactivity score (*p* = 0.020) and CTLA-4+ TILs H. score (*p* = 0.036) were independent prognostic factors affecting overall survival among the other prognostic factors.

**Conclusion:**

PD-L1 and CTLA-4 expression by tumour cells could cooperate with each other in enhancing progression of CRC leading to poor patient outcome, while their expression by TILs could stand against tumour progression.

## Introduction

Colorectal carcinoma (CRC) ranks third and second as regards incidence and mortality, respectively, among the most prevalent cancers, worldwide. In 2020, more than 1.9 million new cases and 935,000 deaths occurred [1]. In Egypt, cancer of rectum and colon represent 3.43% and 2.83% of total malignancies, respectively [2]. There is an increasing incidence of CRC among Egyptian patients, especially those who are ≤ 40 years of age [3].

Tumour-derived immune dysregulation is a key feature of CRC [4]. Increased understanding of the immune tumour microenvironment (TME) allowed investigation of novel immune-based biomarkers and the development of new agents for blockade of immune checkpoint molecules to activate antitumour immunity [5].

Programmed cell death protein 1 (PD-1), an inhibitory checkpoint molecule, belongs to the cluster of differentiation (CD28) family and is expressed on activated T cells’ surface to regulate proliferation and activation. PD-L1 is the dominant ligand for PD-1 and expressed in activated T cells, B cells, dendritic cells, macrophages, endothelial cells and a significant number of tumour cells [6].

Cytotoxic T-lymphocyte-associated protein 4 (CTLA-4) also functions as an immune checkpoint that downregulates immune responses. It is constitutively expressed on dendritic cells, regulatory T cells and competitively binds with the CD80/86 receptor of antigen presenting cells, preventing its interaction with CD28 and blocking the costimulatory signal required for T cell activation [7, 8].

PD-L1 and CTLA-4 immune checkpoint pathways are potent inhibitors of tumour-reactive T cell activation, clonal expansion and subsequent tumour rejection. Their interactions act on inhibiting the activation of T-cells and inducing tumour immune escape. Therefore, they help to create a suitable microenvironment (TME) that enables continuous proliferation of tumour cells [4, 9]. Anti-CTLA-4 and anti-PD-L1 monoclonal antibodies effectively block these pathways resulting in the re-activation and clonal expansion of tumour-reactive lymphocytes. Clinical trials showed remarkable success in patients with different cancers, including melanoma and non-small cell lung cancer (NSCLC) patients [10, 11]. However, reported studies evaluating the prognostic significance of PD-L1 expression in CRC are limited and controversial [6, 12]. To the best of authors’ knowledge, CTLA-4 immunohistochemical expression in CRC has not been investigated.

This study aimed to evaluate the immunohistochemical expression of PD-L1 and CTLA-4 in CRC cases and their relationship with clinicopathological parameters and survival data.

## Materials and methods

This retrospective case–control study included 146 cases divided into: 21 non-neoplastic colonic tissue samples (collected from intussusception, volvulus and diverticulitis), 22 adenoma specimens (collected from colonoscopic biopsies or partial colectomy) and 103 CRC (obtained from surgical colectomy). Formalin-fixed, paraffin embedded tissue blocks were retrieved from the archival material of Pathology Department, Faculty of Medicine, Menoufia University, during the period between 2015 and 2019. The study was approved by Institutional Review Board of Menoufia University. Clinical features, patients’ recurrence and overall survival (OS) data were collected from patients’ medical records. OS data was calculated in months from the date of diagnosis to the time of death or the date of last follow-up visit. Recurrence free survival (RFS) time was calculated from the date of surgery till occurrence of recurrence. The diagnosis of recurrence after surgery was based on typical imaging appearance and confirmed with positive colonoscopy biopsy findings [13].

### Histopathological evaluation

Haematoxylin and eosin (H&E) stained slides were obtained and reassessed according to 2019 WHO classification of tumours of the digestive system [14]. Histopathologic features include: TNM staging [15, 16], Stage grouping, Lymph node ratio (LNR) (rN1: 0% < LNR ≤ 35%, rN2: 35% < LNR ≤ 69%, rN3: LNR > 69%) [17], histopathologic type (Conventional adenocarcinoma, mucinous adenocarcinoma with mucinous component represents >50% and adenocarcinoma with mucinous differentiation ≤50%) [14], tumour grade, lympho-vascular invasion, perineural invasion [14], necrosis, tumour border configuration (TBC) [18], tumour budding (TB) [19], tumour stroma ratio (TSR) [20], intratumoural tumour infiltrating lymphocytes (TILs) [21] and peritumoural TILs [22].

### Tissue microarray construction

H&E-stained slides of the selected cases’ blocks were examined carefully to identify viable and representative areas of each sample. A manual tissue arrayer’s needle (Breecher Instrument, USA) was used to create the tissue microarrays with 2 mm punch size. Three cores of different areas of the tumour and stroma were sampled from each tumour specimen [23].

### Immunohistochemsitry technique

From each tissue microarray (TMA) block, two sections were cut and immunostained using streptavidin-biotin-amplified system. The primary antibodies used were polyclonal rabbit anti-human antibodies including PD-L1 (Conc. and diluted as1:25, Cat#YPA1542) and CTLA-4 (Conc. and diluted as1:100, Cat# YPA1004), both obtained from Chongqing Biospes Co., Ltd, China.

### Interpretation of immunohistochemical results

PD-L1 and CTLA-4 were assessed in TILs as well as epithelial cells and cases were considered as positive when there was brownish cytoplasmic/membranous staining in ≥5% of cells for PD-L1 [24], and in any number of cells for CLTA4 [25]. Furthermore, the following methods were used.

- Intensity was scored from 0 to 3 (0 = Negative, 1 = Mild staining, 2 = Moderate staining and 3 = Strong staining) for both PD-L1 [24, 26] and CTLA-4 [27, 28].
- Percentage: for PD-L1, extent of staining was scored as 0 (<5%), 1 (5%–25%), 2 (26%–50%), 3 (51%–75%) and 4 (>75%) according to the percentage of the positively stained cells [24]. But regarding CTLA-4, positive stromal lymphocytes were recorded as: 0 (0%–25%), 1 (26%–50%), 2 (51%–75%) or 3 (76%–100%) [29] while epithelial cells were not considered due to homogenous staining pattern [28].
- Scoring: for PD-L1, immunoreactivity score (IRS) was obtained by multiplying intensity and percentage, then cases were grouped into Low (≤4) and high (>4) [6, 24, 30]. For CTLA-4, cases were grouped using epithelial intensity score into Low (0, 1) and high (2, 3); and by using stromal H-score into Low (≤mean) and high (>mean) [29].

### Statistical analysis

Data were collected, tabulated and analysed using IBM (Statistical Package for the Social Sciences software package version 20 (Armonk, NY: IBM Corp). Chi-Square (*X*^2^) test was used for qualitative data. Kaplan–Meier curve and log rank test were constructed for survival analysis. Results with *p* ≤ 0.05 were considered as statistically significant.

## Results

### Clinicopathologic data of the studied control and adenoma groups

This study included 21 normal colonic specimens (10 males (47.6%) and 11 females (52.4%), with a mean age of 54.05 years) and 22 colonic adenoma cases (15 males (68.2%) and 7 females (31.8%) with a mean age of 58.91 years, 14 tubulo-villous (63.6%), 5 tubular (22.7%) and 3 villous types (13.6%)). Thirteen of adenoma cases (59.1%) showed low-grade dysplasia and 9 (40.9%) showed high-grade dysplasia.

### Clinicopathologic data of colorectal carcinoma cases

This study included 103 colorectal adenocarcinoma carcinoma cases; their characteristics were demonstrated in Table 1.

### PD-L1 IRS in the studied groups

In non-neoplastic cases, epithelial IRS ranged from 0 to 8 with a mean ± standard deviation (SD) of 2.76 ± 2.72 and 18 specimens (85.7%) showed low IRS. PD-L1+ TILs IRS ranged from 0 to 12 with a mean ± SD of 5.81 ± 4.14 where 14 specimens (66.7%) showed high IRS. In adenoma cases, epithelial IRS ranged from 4 to 12 with a mean ± SD of 6.95 ± 3.32, while PD-L1+ TILs IRS ranged from 0 to 12 with a mean ± SD of 6.91 ± 3.84. Regarding CRC, epithelial IRS ranged from 0 to 12 with a mean ± SD of 5.60 ± 4.62 and 57 cases (55.3%) showed high IRS and PD-L1+ TILs IRS ranged from 0 to 12 with a mean ± SD of 4.45 ± 4.

### Comparison among normal, low-grade adenoma, high-grade adenoma and CRC regarding PD-L1 IRS

**Figure 1.**
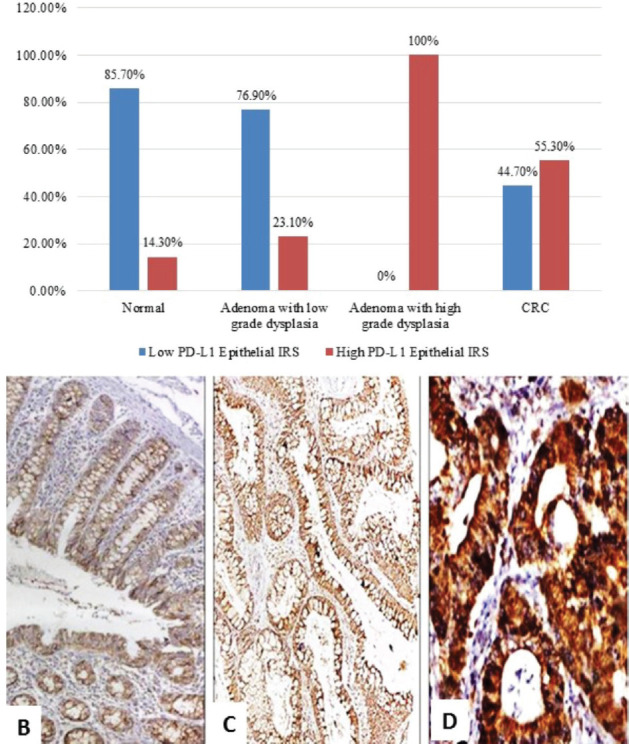
(a): PD-L1 epithelial IRS expression in normal, low-grade adenoma, high-grade adenoma and colorectal adenocarcinoma cases (PD-L1, Programmed cell death ligand-1; IRS, Immunoreactivity score). (b): Normal colonic biopsy showing mild cytoplasmic expression of PD-L1 in both epithelial and stromal lymphocytes (PD-L1 ×40). (c): A case of tubular adenoma showing moderate cytoplasmic expression of PD-L1 in both epithelial and stromal lymphocytes (PD-L1 ×100). (d): A case of colonic adenocarcinoma showing strong cytoplasmic expression of PD-L1 in malignant cells (PD-L1 ×200).

There was a significant difference between the four groups regarding PD-L1 epithelial IRS (*p* ˂ 0.001). High PD-L1 epithelial expression was significantly in favour of CRC (*p* ˂ 0.001) and adenoma with high-grade dysplasia (*p* ˂ 0.001) compared to normal specimens. Additionally, high PD-L1 epithelial expression was significantly in favour of adenocarcinoma compared to adenoma with high-grade dysplasia (*p* = 0.009). On the other hand, no statistical difference was detected regarding PD-L1+ immune cells among the three groups (Figure 1).

### Relationship between immunohistochemical epithelial expression of PD-L1 and clinicopathological parameters of CRC cases

**Figure 2.**
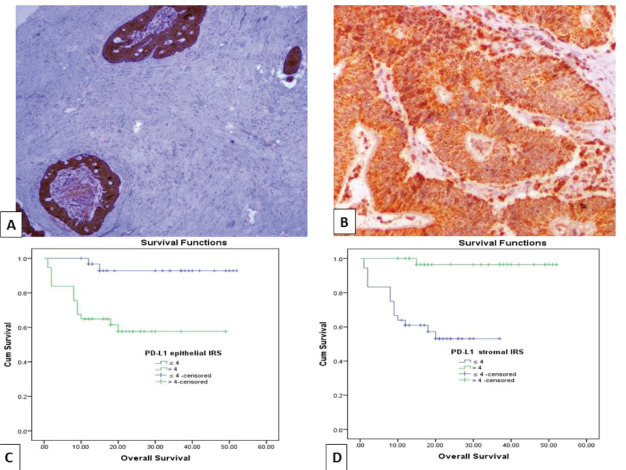
(a): Colonic adenocarcinoma with low TSR showing high PD-L1 IRS in malignant cells and low IRS of PD-L1^+^ stromal cells (PD-L1 ×100). (b): Low-grade colonic adenocarcinoma with high TSR, showing high PD-L1 IRS in malignant cells and PD-L1+ stromal cells (PD-L1 ×200). (c and d): Kaplan–Meier survival curve demonstrating the impact of PD-L1 epithelial IRS (*p* value = 0.001) and PD-L1+ stromal cells IRS (*p* value < 0.001) on OS. (PD-L1, Programmed cell death ligand 1; IRS, Immunoreactivity score).

High PD-L1 epithelial IRS showed significant association with large tumour size (*p* = 0.038), presence of perforation (*p* = 0.023), advanced pathologic T stage (*p* = 0.007), infiltrative TBC (*p* ˂ 0.0001), high TB score (*p* = 0.004), low TSR (*p* = 0.012) and absence of cap like peritumoural lymphocytic reaction (*p* = 0.036). While, high IRS of PD-L1+ TILs showed a significant relationship with absence of perforation (*p* = 0.029), early pathologic T stage (*p* = 0.019), pushing TBC (*p* ˂ 0.0001), lower TB score (*p* = 0.002), high TSR (*p* = 0.017) (Figure 2a) and presence of cap like peritumoural lymphocytic reaction (*p* = 0.049) (Figure 2b) (Table 2). Also, inverse relationship has been detected between PD-L1 epithelial IRS and PD-L1+ TILs IRS (*p* > 0.001).

### CTLA-4 assessment score in the studied groups

In non-neoplastic group, most cases (17 (81.0%)) showed low epithelial CTLA-4 intensity score and 15 cases (71.4%) showed low H. score in stromal lymphocytes with a mean ± SD of 6.19 ± 2.02. In adenoma group, 11 specimens (50%) showed high epithelial intensity score, while 13 specimens (49.9%) showed high H. score in stromal lymphocytes with a mean ± SD of 5.82 ± 2.02. Most of adenocarcinoma cases (69 (67%)) showed high epithelial intensity score and 49 cases (50.5%) showed high H. score in stromal lymphocytes with a mean ± SD of 4.59 ± 3.16.

### Comparison among normal, low-grade adenoma, high-grade adenoma and CRC regarding CTLA-4

**Figure 3.**
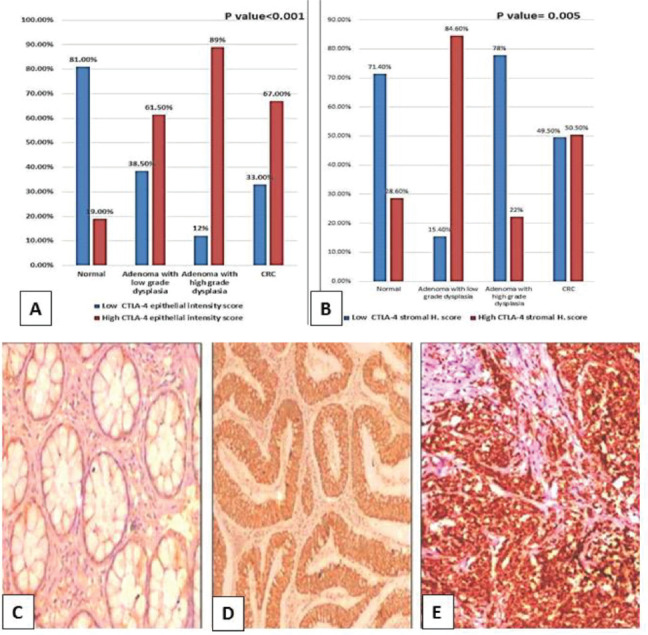
(a): Epithelial intensity score and (b): Lymphocytic CTLA-4 in normal, low-grade adenoma, high-grade adenoma and adenocarcinoma cases. (CTLA-4, Cytotoxic T-lymphocyte-associated protein 4; CRC, Colorectal adenocarcinoma; H. score, Histoscore). (c): Normal colonic biopsy showing mild cytoplasmic expression of CTLA-4 in both epithelial and stromal lymphocytes (CTLA-4 ×200). (d): Tubular adenoma showing moderate cytoplasmic expression of CTLA-4 in epithelial cells and mild cytoplasmic expression in stromal lymphocytes (CTLA-4 ×200). (e): High-grade colonic adenocarcinoma showing strong cytoplasmic expression of CTLA-4 in malignant cells (CTLA-4 ×200).

There was a significant difference regarding both epithelial CTLA-4 (*p* ˂ 0.001) and CTLA-4+ TILs (*p* = 0.005). High CTLA-4 epithelial expression was significantly in favour of adenocarcinoma (*p* ˂ 0.001), adenoma with high-grade dysplasia (*p* value ˂ 0.001) and adenoma with low-grade dysplasia (*p* = 0.011) compared to normal specimens (Figure 3). However, high CTLA-4+ TILs was significantly in favour of adenoma with low-grade dysplasia compared to normal specimens (*p* value = 0.001), high-grade dysplasia (*p* = 0.003) and adenocarcinoma cases (*p* = 0.019).

### Relationship between CTLA-4 expression and clinicopathological parameters in studied CRC cases

**Figure 4.**
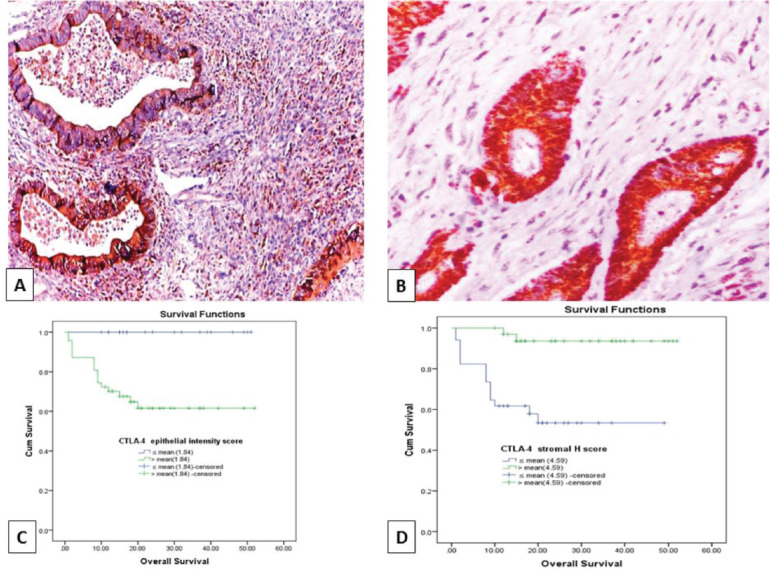
(a): Colonic adenocarcinoma with pushing tumour margin, showing high H-score of CTLA-4+ TILs and high CTLA-4 intensity score in malignant cells (CTLA-4 ×100). (b): low-grade colonic adenocarcinoma with no peritumoural lymphocytic reaction showing high CTLA-4 intensity score in malignant cells and low H- score of CTLA-4+ TILs (CTLA-4 ×200). (c and d): Kaplan–Meier survival curve demonstrating the impact of CTLA-4 epithelial intensity score (*p* value = 0.003) and CTLA-4+ TILs H score (*p* value < 0.001) on OS. (CTLA-4, Cytotoxic T lymphocyte-associated antigen-4 (CTLA-4); H score, Histoscore; TILs, Tumour infiltrating lymphocytes).

High CTLA-4 epithelial intensity was significantly associated with positive LN involvement (*p* = 0.020), presence of an infiltrative TBC (*p* ˂ 0.001) and absence of cap like peritumoural lymphocytic reaction (*p* = 0.05) (Figure 4a). While low H. score of CTLA-4+ TILs showed a significant association with advanced pathologic T stage (*p* = 0.044), increased number of positive LNs (*p* = 0.046) and infiltrating TBC (*p* = 0.004) (Figure 4b) (Table 3). Furthermore, there was a significant inverse relationship between CTLA-4 epithelial intensity score and CTLA-4+ TILs H. score (*p* > 0.001).

### Relationship between PD-L1 expression and CTLA-4 expression in CRC cases

PD-L1 epithelial IRS showed a positive relationship with CTLA-4 epithelial intensity score (*p* < 0.001) and an inverse relationship with CTLA-4+ TILs H. score (*p* < 0.001). However, high scores of PD-L1+ TILs showed a significant association with CTLA-4+ TILs in CRC cases (*p* < 0.001) and an inverse relationship with CTLA-4 epithelial intensity score (*p* < 0.001) (Table 4).

### The impact of PD-L1 and CTLA-4 immuno-expression on OS and RFS of CRC patients

OS data was available for 68 cases (66%). Their survival time ranged from 1 to 52 months with a mean ± SD of 21.47 ± 13.52 months and a median of 18 months. While 14 cases (20.6%) experienced tumour recurrence in a period ranged from 1 to 52 months with a mean ± SD of 19.59 ± 14.31 and a median of 16 months. Prolonged OS was significantly associated with low PD-L1 epithelial IRS (*p* = 0.001), high PD-L1+ TILs IRS (*p* ˂ 0.001) (Figure 2c and d), low CTLA-4 epithelial intensity score (*p* = 0.003) and high CTLA-4+ TILs H. score (*p* ˂ 0.001) (Figure 4c and d). Moreover, short RFS was significantly associated with high PD-L1 epithelial IRS (*p* = 0.005) and high CTLA-4 epithelial intensity score (*p* = 0.012). By multivariate Cox regression analysis, PD-L1+ TILs IRS (*p* = 0.020) and CTLA-4+ TILs H. score (*p* = 0.036) were independent prognostic factors affecting OS among the other prognostic factors.

## Discussion

In current study, PD-L1 epithelial expression increased significantly in adenoma with high-grade dysplasia and adenocarcinoma cases when compared to normal specimens (*p* ˂ 0.001). This agreed with Miller *et al* [31] who reported an increase in expression of PD-L1 in different stages of progression from dysplasia to invasive malignancy in rectal lesions. This can be explained by presence of inherent tumoural signalling pathways, like *PTEN* gene deletion, which often leads to the over-activation of the phosphatidylinositol 3 kinase (PI3K)/Protein kinase B (AKT) signalling pathway, thus increasing PD-L1 expression, which will inhibit activation of cytotoxic T cells [32]. Moreover, interferon-γ (IFN)-γ secreted by cells participating in tumour elimination (i.e. CD4+ T helper 1 cells and activated T cells) may induce high PD-L1 expression in tumour cells [33].

Also, high PD-L1 expression in adenocarcinoma was associated with poor prognostic parameters such as large tumour size (*p* = 0.038), presence of perforation (*p* = 0.023) and advanced pathologic T stage (*p* = 0.007). This may be a downstream effect of PD-L1 interactions with its receptor PD-1, transmitting a negative signal to T cell-mediated immune responses (priming, growth, proliferation and functional maturation), thus enhancing tumour progression [34]. This is supported by Hacking *et al* [35] who found high PD-L1 expression in CRC cases with high mitotic index and bad patient outcome. High PD-L1 epithelial expression showed a statistically significant relationship with low peritumoural lymphocytic reaction (*p* = 0.036). This could be explained by the role of the abnormal activation of the PD-L1/PD-1 signalling pathway in inhibiting the proliferation and differentiation of T cells by different mechanisms, and induction of T cell apoptosis to enable tumour immune evasion [33]. This was supported by Shan *et al* [34] who showed that PD-L1 positive tumour tissues were correlated with low-density of TILs. On the other hand, Droeser *et al* [9] found CD8+ T cell infiltration was unexpectedly increased in CRC cases with high PD-L1 expression suggesting these T cell population did not express PD-1, so they were not under the inhibitory effect of PD-L1/PD-1 signalling pathway.

Remarkably, high PD-L1 epithelial expression was significantly associated with infiltrative TBC (*p* ˂ 0.0001) and high TB score (*p* = 0.004) that reflect epithelial–mesenchymal-transition (EMT) profile of the tumour. Therefore, the current study suggests that PD-L1 upregulation in cancer may not be only mediated by the inhibitory effect on T cell immune response, but also through acting on EMT. Martinez-Ciarpaglini *et al* demonstrated that cases expressing high PD-L1 were associated with increased expression of the EMT programme markers and high TB score [36]. Interestingly, high PD-L1 epithelial expression was significantly correlated to low TSR (high stroma percent) (*p* = 0.012) and this was explained as high stroma might promote PD-L1 expression by tumour cells through releasing proinflammatory molecules or cytokines, such as (IFN)-γ, IFN-α, IFN-β, tumour necrosis factor (TNF)-α, epidermal growth factor, interleukin (IL)-17, IL-4 and IL-27 [37]. In lung cancer, findings showed that stromal derived factors, such as transforming growth factor (TGF)-β mediates PD-L1 epithelial expression through an epigenetic mechanism by DNA methylation [38]. Furthermore, it has been suggested that stromal fibroblasts mediated the expression of PD-L1 by the adjacent cancer cells [11]. Furthermore, univariate analysis showed that cases with high PD-L1 expression in CRC cells were significantly associated with short OS (*p* = 0.001) and short RFS (*p* = 0.005). These associations were explained by the role of PD-L1 in tumour immune escape mechanisms, tumour progression, aggressiveness and invasion which correlate with poor patient outcome [39].

On the other hand, Droeser *et al* [9] reported that cases with high PD-L1 expression in CRC cells were positively correlated with improved OS. However, this was only noticed in mismatch repair proficient CRC group. The study hypothesised this could be related to the concomitant increase in CD8+ T cell infiltration and IFN-c producing T cells which mirror T cell activation in those cases. Regarding TILs, high PD-L1 expression was observed in 43.7% of studied CRC cases. High PD-L1+ TILs expression showed a significant relationship with favourable prognostic parameters as absence of perforation (*p* = 0.029) and early pathologic T stage (*p* = 0.019). These associations were also reported by Kim *et al* [40] in CRC suggesting that high PD-L1 expres sion in stromal lymphocytes reflects presence of high density of stromal lymphocytes which represent the immune response that stands against tumour progression.

Furthermore, high PD-L1+ TILs expression showed a highly significant association with pushing TBC (*p* ˂ 0.0001) and lower TB (*p* = 0.002). These results agreed with Martinez-Ciarpaglini *et al* who reported that all cases of CRC with low-grade TB and/or pushing TBC showed higher PD-L1 expression in peritumoural lymphocytes [36]. The anti-tumour immune response resulting from upregulation of PD-L1 positive stromal lymphocytes eliminates the invasive power of tumour presented in TB [36]. However, Valentini *et al* [41] showed that pattern of advancing border and TB were not associated with PD-L1 expression in stromal lymphocytes.

Remarkably, high PD-L1+ TILs expression was significantly associated with high peritumoural lymphocytic reaction (*p* = 0.049), explained by the vital impact of stromal PD-L1 on T cells survival and proliferation during immune response. For T cells with low expression of PD-L1, after antigen-initiated expansion, they underwent more apoptosis *in vitro* [42]. These finding was also in agreement with Lee *et al* [43], Kim *et al* [40] but, against O’Malley *et al* [44] who suggested a role of PD-L1 positive mesenchymal stromal cells in suppressing CD8+ antitumour immune proliferation. Additionally, the present results demonstrated that low PD-L1+ TILs expression was significantly correlated to low TSR (*p* = 0.017) and this is explained as secretion of PD-L1 by mesenchymal stromal cells could suppress T cell proliferation so downregulates T cell surface PD-L1 expression [45].

Univariate analysis showed that high PD-L1+ TILs expression was significantly associated with prolonged OS (*p* ˂ 0.001) and prolonged RFS (*p* = 0.002). This agreed with Kim *et al* [40] who reported that the good prognostic outcome in CRC cases with high PD-L1 stromal expression may be due to the inverse relationship between PD-L1 stromal and epithelial expression levels, due to escape from immunosuppressive impact of PD-1/PD-L1 pathway, thereby they had better outcome. Moreover, the inverse relationship between PD-L1 expression in CRC cells and PD-L1+ TILs observed in the current study (*p* < 0.001) agreed with Kim *et al* [40] who showed the inhibitory effect that PD-L1 expression in CRC cells exerted on TILs through inhibiting their proliferation and inducing apoptosis, thus reducing their density.

The current study demonstrated a significant increase in CTLA-4 epithelial expression in adenoma with low-grade dysplasia, high-grade dysplasia and adenocarcinoma compared to normal specimens (*p* ˂ 0.001). These findings agreed with Xiang *et al* [46] showing that CTLA-4 was upregulated in gastric cancer tissue when compared to normal mucosa. This may represent a central role of CTLA-4 expression in tumour progression cycle through inducing tumour immune escape [47]. Additionally, CTLA-4 promotes the expression of Casitas-B-lineage lymphoma (Cbl)-b protein or suppress the formation of zeta-associated protein 70 to negatively regulate T cell activation [48].

To the authors’ knowledge, CTLA-4 immunoexpression and its correlation with clinicopathological parameters in CRC were not widely studied. High CTLA-4 epithelial expression showed a significant relationship with an infiltrative TBC (*p* = 0.001) which is explained as CTLA-4 expression correlates with invasive tumour power [28, 49–52].

Furthermore, high CTLA-4 expression was significantly associated with low peritumoural lymphocytic reaction (*p* = 0.05). An essential step in T-cell activation process is a costimulatory signal elicited by the engagement of CD28 on T cells with B7 ligands (CD80 and CD86) on antigen presenting cells. However, in cancer, the CD28 homologue CTLA-4 is translocated from intracellular storage to the plasma membrane of T cells, competitively binds to B7 ligands on antigen presenting cells (APCs) with higher affinity, thereby preventing CD28-mediated T cell activation. Moreover, CTLA-4 attenuates the T cell response through the inhibition of IL-2 and blockage of cell cycle progression [53, 54].

High CTLA-4 epithelial expression was significantly correlated with short OS (*p* = 0.003) and short RFS (*p* = 0.012) of adenocarcinoma cases. In line with our results, Santoni *et al* [52] reported similar findings in thymoma cases stating that tumour-associated CTLA-4 could promote tumour progression and poor outcome by inhibiting the anti-tumour T cell immunity and inducing tumour-specific T cell apoptosis. Moreover, high CTLA-4 expression was associated with short OS and disease-free survival of breast cancer cases [55] and NSCLC [56] due to downregulation of effector T-cell function and upregulation of regulatory immunosuppressive T-cell.

High CTLA-4+ TILs expression was in favour of adenoma with low-grade dysplasia when compared to adenoma with high-grade dysplasia and adenocarcinoma cases. These findings agreed with Yu *et al*. [55] who found that the anti-tumour immune response resulting from high density of CTLA-4+ lymphocytes in early precursor lesions, compared to advanced stages of breast cancer. Also, high CTLA-4+ TILs expression was associated significantly with early pathologic T stage (*p* = 0.044), decreased number of positive LNs (*p* = 0.046) and pushing TBC (*p* = 0.004). High CTLA-4+ TILs expression represents a mirror image to high density of stromal lymphocytes which help in tumour elimination; therefore, it is associated with pathological features of low tumour progression potential [7, 28]. Low CTLA-4+ TILs showed a significant association with short OS (*p* ˂ 0.001) and short RFS (*p* = 0.008). In line with the current results, high stromal CTLA-4 expression independently predicted prolonged survival in NSCLC cases [28] and breast cancer [55]. The positive association between stromal CTLA-4 and survival was explained by the presence of a TME in which anti-tumour immunity properties dominate [28].

Moreover, the current results showed an inverse relationship between CTLA-4 epithelial and TILs’ expression (*p* ˂ 0.001). Interestingly, Yu *et al* reported that the good prognostic effect of high CTLA-4 lymphocytic expression was noticed only when tumour CTLA-4 expression was low [55]. Thereby, these cases were not under the immune suppressive effect of tumour-associated CTLA-4 expression [55]. Moreover, the present results detected a positive relationship between PD-L1 expression and CTLA-4 expression in tumour cells (*p* < 0.001). This finding was supported by the synergistic effect of using dual blockade compared with single-agent blockade in enhancing antitumour immune response as reported in preclinical studies and initial clinical trials because they interact in complementary pathways [57].

## Conclusion

PD-L1 and CTLA-4 expression by tumour cells in CRC could cooperate with each other in enhancing tumour progression leading to poor patient outcome. However, their expression by TILs could stand against tumour progression. There is an interaction and crosstalk between the neoplastic cells and TILs, manifested by their enhancing or inhibitory impact on PD-L1 and CTLA-4 release. Enhanced understanding of the mechanisms underlying PD-L1 and CTLA-4 interactions in CRC cases is needed. Inhibition of PD-L1 and CTLA-4 immune checkpoints is recommended as a type of target immunotherapy in CRC patients.

## Declaration of interest

None.

## Funding statement

This research did not receive any specific grant from funding agencies in the public, commercial or not-for-profit sectors.

## Conflicts of interest/competing interests

The authors have no conflicts of interest to declare.

## Availability of data and material (data transparency)

The data analysed during the current study are available from the corresponding author on reasonable request.

## Figures and Tables

**Table 1.** Clinicopathological data of colorectal adenocarcinoma cases.

Variables		Colorectal adenocarcinoma cases	
		*N*	%
Age	≤55 years (mean)	40	38.8
	>55 years (mean)	63	61.2
Gender	Male	37	35.9
	Female	66	64.1
Tumour location	Proximal colon	38	36.9
	Distal colon	39	37.9
	Rectal	26	25.2
Tumour size (cm)	≤5.98 (mean)	51	49.5
	>5.98 (mean)	52	50.5
Gross morphology	Fungating mass	54	52.4
	Ulcerative	17	16.5
	Infiltrative	32	31.1
Gross perforation	Yes	16	15.5
	No	87	84.5
T stage	Early	25	24.3
	Advanced	78	75.7
N stage	Negative LN (No) Positive LN (N1 & N2)	56 47	54.4 45.6
M stage	Mx	35	34
	M0	60	58.2
	M1	8	7.8
Stage grouping	Early (I & II) Advanced (III & IV)	38 30	55.9 44.1
N. investigated LNs	≤14.92 (mean)	61	59.2
	>14.92 (mean)	42	40.8
N. positive LNs	≤1.78 (mean) >1.78 (mean)	72 31	69.9 30.1
LNR	rN1 rN2 rN3	88 10 5	85.4 9.7% 4.9
Histopathologic type	Conventional	72	69.9
	Mucinous	14	13.6
	Adenocarcinoma with mucinous differentiation	17	16.5
Tumour grade	High grade	24	23.3
	Low grade	79	76.7
Lymphovascular invasion	Positive	31	30.1
	Negative	72	69.9
Perineural invasion	Positive	20	19.4
	Negative	83	80.6
Necrosis	Present	22	21.4
	Absent	81	78.6
TBC	Pushing	34	33.0
	Infiltrating	69	67.0
TB score	Low	45	43.7
	Intermediate	35	34.0
	High	23	22.3
TSR	High	67	65.0
	Low	36	35.0
Intratumoural infiltrating lymphocytes percent	≤24.42 (mean)	56	54.4
	>24.42 (mean)	47	45.6
Peritumoural TILs (Jass score)	No cap like	31	30.1
	Cap like	72	69.9

*N*, Number; SD, Standard deviation; LNs, Lymph nodes; %, Percent; CRC, Colorectal cancer

**Table 2.** Relationship between PD-L1 epithelial and stromal immune cells IRS with clinicopathological data of adenocarcinoma cases.

Variables		PD-L1 epithelial IRS				Test *p*. value	PD-L1+ TILs IRS				Test *p*. value
		Low ≤ 4 (*N* = 46)		High > 4 (*N* = 57)			Low ≤ 4 (*N* = 58)		High > 4 (*N* = 45)		
		*N*	%	*N*	%		*N*	%	*N*	%	
Tumour size (cm)	≤5.98 (mean)	28	54.9	23	45.1	*X^2^ =* 4.28 *p =* 0.038	24	47.1	27	52.9	*X^2^ =* 3.51 *p =* 0.061
	>5.98 (mean)	18	34.6	34	65.4		34	65.4	18	34.6	
Perforation	Yes	3	18.8	13	81.2	*X^2^ =* 5.14 *p =* 0.023	13	81.2	3	18.8	*X^2^ =* 4.7 *p =* 0.029^a^
	No	43	49.4	44	50.5		45	51.7	42	48.3	
T stage	Early	17	68	8	32	*X^2^ =* 7.27 *p =* 0.007	9	36	16	64	*X^2^ = 5.3* *p =* 0.019^a^
	Advanced	29	37.2	49	62.8		49	62.8	29	37.2	
	Low grade	38	41.9	41	58.1		41	52	38	48	
TBC	Pushing	24	70.6	10	29.4	*X^2^ =* 13.80 *p* ≤ 0.001^b^	10	29.4	24	70.6	*X^2^ =* 14.92 *p* ≤ 0.001^b^
	Infiltrating	22	31.9	47	68.1		48	69.7	21	30.4	
TB score	Low	28	62.2	17	37.8	*X^2^ =* 11.31 *p =* 0.004^b^	17	37.8	28	62.2	*X^2^ =* 12.04 *p =* 0.002^b^
	Intermediate	13	37.1	22	62.9		23	65.7	12	34.3	
	High	5	21.7	18	78.3		18	78.3	5	21.7	
TSR	High	36	76.6	31	23.4	*X^2^ =* 6.38 *p =* 0.012^a^	32	47.8	35	52.3	*X^2^ =* 5.69 *p =* 0.017^a^
	Low	10	27.8	26	72.2		26	72.2	10	27.8	
Peritumoural TILs (Jass score)	No cap like	9	29	22	71	*X^2^ =* 4.38 *p =* 0.036^a^	22	71	9	29	*X^2^ =* 3.87 *p =* 0.049^a^
	Cap like	37	51.4	35	48.6		36	50	36	50	

*N*, Number; SD, Standard deviation; %, Percent; LNs, Lymph nodes; PD-L1, Programmed cell death ligand 1; IRS, Immunoreactivity score; TILs, Tumour infiltrating lymphocytes; *X*² = Chi square

a significant

b highly significant

**Table 3.** Relationship between CTLA-4 epithelial intensity score and CTLA-4 TILs H-score with clinicopathological data of adenocarcinoma cases.

Variables		CTLA-4 epithelial intensity score				Test *p* value	CTLA-4+ TILs H. score				Test *p* value
		Low (0, 1) (*N* = 34)		High (2, 3) (*N* = 69)			Low ≤ 4.59 (mean) (*N* = 51)		High > 4.59 (mean) (*N* = 52)		
		*N*	%	*N*	%		*N*	%	*N*	%	
T stage	Early	11	44	14	56	*X^2^ =* 1.803 *p =* 0.179	8	32	17	68	*X^2^ =* 4.05 *p =* 0.044^a^
	Advanced	23	29.5	55	70.5		43	55.1	35	44.9	
N stage	Negative LN Positive LN	24 10	42.9 21.3	32 37	57.1 78.7	*X^2^ =* 5.38 *p =* 0.020^a^	23	41.1	33	58.9	*X^2^ =* 3.49 *p =* 0.061
							28	59.6	19	40.4	
TBC	Pushing	19	55.9	15	44.1	*X^2^ =* 12.007 *p* ≤ 0.001^b^	10	29.4	24	70.6	*X^2^ =* 8.205 *p =* 0.004^b^
	Infiltrating	15	21.7	54	78.3		41	59.4	28	40.6	
Peritumoural TILs (Jass score)	No cap like	6	19.4	25	80.6	*X^2^ =* 3.739 *p =* 0.05^a^	19	61.3	12	38.7	*X^2^ =* 2.46 *p =* 0.117
	Cap like	28	38.9	44	61.1		32	44.4	40	55.6	
N. positive LNs	≤1.78 (mean)	29	40.3%	43	59.7%	*X^2^ =* 2.201 *p =* 0.333	31	43.1	41	56.9	*X^2^ =* 3.992 *p =* 0.046^a^
	>1.78 (mean)	5	16.1	26	83.9		20	64.5	11	35.5	

*N*, Number; SD, Standard deviation; %, Percent; LNs, Lymph nodes; CTLA-4, Cytotoxic T lymphocyte-associated antigen-4; *X*², Chi square

a significant

b highly significant

**Table 4.** Relationship between PD-L1 expression and CTLA-4 expression in CRC cases.

Variables		PD-L1 epithelial IRS				Test *p* value	PD-L1^+^ TILs IRS				Test *p* value
		Low ≤ 4 (*N* = 46)		High > 4 (*N* = 57)			Low ≤ 4 (*N* = 58)		High > 4 (*N* = 45)		
		*N*	%	*N*	%		*N*	%	*N*	%	
CTLA-4 epithelial intensity score	Low (0, 1) (*N* = 34)	29	85.3	5	14.7	*X^2^ =* 33.90 *p <* 0.001	5	14.7	29	85.3	*X^2^ =* 35.71 *p <* 0.001
	High (2, 3) (*N* = 69)	17	24.6	52	75.4		53	76.8	16	23.2	
CTLA-4+ TILs H. score	Low ≤ 4.59 (mean) (*N* = 51)	4	7.8	47	92.2	*X^2^ =* 55.40 *p* ≤ 0.001	45	88.2	6	11.8	*X^2^ =* 41.84 *p* ≤ 0.001
	High > 4.59 (mean) (*N* = 52)	42	80.8	10	19.2		13	25.0	39	75.0	

PD-L1, Programmed cell death ligand 1; IRS, Immunoreactivity score;

CTLA-4, Cytotoxic T-lymphocyte-associated protein 4; H.score, Histoscore; TILs, Tumour infiltrating lymphocytes
